# Patient Profiles in a High Secure Prison Mental Health Unit: Six Years of Australian Data

**DOI:** 10.1192/j.eurpsy.2026.10948

**Published:** 2026-07-31

**Authors:** W. Rienecker, N. Yee, K. Dean

**Affiliations:** 1 Discipline of Psychiatry and Mental Health, School of Clinical Medicine, University of New South Wales; 2 Justice Health and Forensic Mental Health Network, Sydney, Australia

## Abstract

**Introduction:**

The high prevalence of mental illness in prisons is well-established. Yet, the provision of mental health care in prisons, including involuntary treatment, remains controversial. Ongoing discourse argues that those with serious mental illness in prisons should be treated in specialised mental health facilities. Conversely, specialist prison ‘hospitals’ or prison mental health units have been developed in many jurisdictions to provide more intensive mental health assessment and support to those in custody. However, little is known about these specialist units, including their patient populations and outcomes.

**Objectives:**

The present study examined the sociodemographic, clinical and offending characteristics of a cohort of adult male patients admitted to a specialist prison mental health unit, the Long Bay Hospital Mental Health Unit (LBH-MHU) in Sydney, Australia. The LBH-MHU is the only facility in the country which permits involuntary mental health treatment within a custodial environment.

**Methods:**

Key sociodemographic, clinical, offence-related and length of stay variables were extracted from routinely recorded data for people who were admitted to the LBH-MHU between the years 2016 to 2021. Descriptive statistics were obtained for the entire sample, and subgroup comparisons were separately examined for First Nations and Culturally and Linguistically Diverse (CALD) patients using binary logistic regression and Mann-Whitney U tests.

**Results:**

A total of 477 men were admitted to the LBH-MHU over the six years with a median age of 36.7 years at time of admission. The average length of stay was 90 days. The majority of the patients had a schizophrenia spectrum disorder diagnosis (83.4%) and a violent index charge (74.5%) associated with their current custodial episode. First Nations patients (*Mdn* = 32.7 years) were significantly younger than non-First Nations patients (*Mdn* = 37.6 years), whereas CALD patients did not significantly differ from non-CALD patients.

**Conclusions:**

The present study offers unique insights into the characteristics of patients admitted into a specialist, high-secure, prison mental health unit in Australia. The current findings highlights opportunities to improve mental health care provision within custodial settings and the need for alternative pathways for those with a serious mental illness in prisons, including transfers to specialised forensic mental health facilities.

**Disclosure of Interest:**

None Declared

